# Hydropower-induced selection of behavioural traits in Atlantic salmon (*Salmo salar*)

**DOI:** 10.1038/s41598-021-95952-1

**Published:** 2021-08-12

**Authors:** Tormod Haraldstad, Thrond O. Haugen, Esben M. Olsen, Torbjørn Forseth, Erik Höglund

**Affiliations:** 1 Norwegian Institute for Water Research, Jon Lilletuns vei 3, NO-4879 Grimstad, Norway; 2 Department of Ecology and Natural Resource Management, Norwegian University of Life Sciences, NO-1432 Ås, Norway; 3 Institute of Marine Research, Flødevigen, NO-4817 His, Norway; 4 Norwegian Institute for Nature Research, NO-7485 Trondheim, Norway

**Keywords:** Ecology, Animal migration, Behavioural ecology, Conservation biology, Freshwater ecology

## Abstract

Renewable energy projects such as hydropower facilities contribute towards meeting the world`s growing energy demands and urgent need for mitigating climate change. However, such infrastructure has the potential to substantially alter the environment which, in turn, can induce new challenges related to for instance fish migration conditions. As a consequence, local adaptations related to pre-development migration conditions may be affected for influenced populations. To explore selection regimes operating at a river hydropower plant, we monitored Atlantic salmon smolt individuals during their seaward migration. When passing the hydropower plant, the smolts chose between a surface fish passage or a submerged turbine intake. Smolts were scored for behavioural type (basal locomotor activity, net restrain (a measure of escape responses) and willingness to leave a familiar environment) prior to their migration choice, and we found that smolts with high basal activity had higher probability of using the fish passage than the turbine intake. In addition, migration route choice was a partly consistent trait in that fish that had previously passed a hydroelectric facility by using a fish passage rather than the turbine intake were significantly more likely to use it again when faced with the same choice. Higher mortality among turbine migrants could potentially reduce or eliminate particular behaviour types within populations- and the corresponding population genetic diversity that is essential to cope with future environmental challenges.

## Introduction

Human activities, such as intensive agriculture, forest management and urbanization have tremendous impact on the natural world and have thus been changing the adaptive landscape of many organisms. Industrialisation, including overfishing, release of pesticides, herbicides and release of toxic compounds, are well known examples of such human induced selection. Accordingly, humans may be the most powerful evolutionary force currently acting on wild animals ^1^, and therefore a central driving force in contemporary evolution^2,3^.

Renewable energy is vital for meeting the worlds growing energy demands and urgent need to mitigate climate change. This type of energy source stands in contrast to fossil fuels, which are being used more quickly than they are being replenished. However, the local ecological impacts of such hydro, wind and solar technologies can be detrimental^4^. Hydropower and its associated infrastructure have large impacts on the local environments. Reservoirs can lead to habitat loss and fragmentation of both aquatic and terrestrial animal populations ^5^, while run-of-the-river hydropower plants have the potential to substantially change the aquatic environment by altering the flow regimes and disrupting connectivity in the river^6,7^. The latter includes halting up- and downstream migration of fish. In particular, downstream migration through hydropower turbines are associated with high fish mortality^8^. As such, hydropower dams are considered one of the main challenges for restoring and maintaining sustainable migratory fish populations worldwide ^4,9,10^. However, surprisingly little attention has been paid to the important question of whether hydropower may also induce new selection regimes that affect fish populations.

Atlantic salmon (*Salmo salar*) is an iconic species, with high ecological and socioeconomic importance. Its anadromous life history requires unimpeded migration routes between the species` freshwater nursery and spawning habitats and oceanic feeding areas^11^. Thus, hydropower dams are major obstacles for Atlantic salmon populations^12,13^. A range of mitigation measures have been explored for fish passage, often exploiting fish behaviour patterns to divert fish away from dangerous routes and guide or attract fish towards alternative migration paths^14^. Historically, attention has been directed towards implementing measures to assist upstream spawning migrants, and mitigation measures for descending smolts were scarce. Mortality of smolts migrating through hydropower turbines has been documented from a variety of rivers and for various turbine types^15–17^. More recently, measures to facilitate downstream migrating smolts has attained increased focus ^18,19^. The small body size of Atlantic salmon smolts and their tendency to follow the main current, impose design challenges to measures that prevent migration into turbines. Investigations of fish passage efficiency from a range of sites reveals a general low overall efficiency ^20^. Individuals may not encounter, locate or respond to guidance structures at hydropower facilities during their downstream movements leading to the majority of fish instead using the turbine intake as a migration route^21^, which may lead to high mortalities ^15,17^.

It is widely recognised that fish passages can be species^20,22,23^ and size selective ^24–26^. For instance, a size-selective fish passage, led to loss of the largest size classes of Atlantic salmon spawners in Penobscot River, US ^26^. By contrast, construction of a fish ladder in the river Gudbrandsdalslågen, Norway led to stabilizing body-size selection favouring midsized brown trout (*Salmo trutta*) in contrast to the pristine waterfall that induced directional selection, favouring larger individuals ^24^. Even within the same size-group of brown trout, upstream fish passage may induce selection on particular life-history phenotypes, e.g., favouring anadromous phenotypes at the expense of freshwater-resident adults ^27^. Similar selective regimes are expected to occur at fish passage guidance structures for descending smolts, however, studies that address this issue are still lacking. Haraldstad ^28^ suggested that the migration route choice of salmonid smolts at a hydropower forebay was not random, but rather a consequence of individual differences in physiological and behavioural traits. However, it remained to be investigated whether the significant differences in mortality related to migration-route choice at hydropower dams would induce selection on individual behavioural traits.

It is widely recognized that individuals from the same population often cluster into behaviour types, or personalities ^29^. Such personalities typically have a significant heritable component, meaning that populations can be expected to evolve (i.e., a genetic change) in response to selection acting directly or indirectly on these personalities ^30^. Furthermore, trait values that derive from behavioural assay tests are often highly associated with both physiological traits and fitness-related traits like growth, maturation, and reproduction, which again forms the basis for pace-of-life syndromes (POLS) ^31^. From a population of descending salmonid smolt perspective the choice of a migration route may therefore not only have instant survival consequences, but also long-term adaptational consequences. If part of the route choice variation is coupled to variation in individual behavioural types, and part of this individual variation is heritable, the among-trait genetic correlation structure associated with potential pace-of-life syndrome and the strength and consistency of the selection involved will to a large extent determine to what degree the route choice has long-term adaptational consequences for the population.

To explore potential selection regimes at a hydropower plant, we compared the migration route of behaviour-scored Atlantic salmon smolt individuals and their link to individual choices between migrating through a surface-layer fish passage and a submerged turbine intake under a wide range of environmental conditions. All study individuals were wild-caught descending smolts, that were tagged (Passive Integrated Transponders, PIT) and subjected to three behavioural assays described below; basal locomotor activity, net restrain and willingness to leave a familiar environment. After the assays were completed, the fish were released in a hydropower forebay and the individual migration route choices were then recorded from route-specific trap recaptures. Moreover, the consistency of migration-route choice was investigated by comparing two groups of smolts; one “naive” group, never confronted with the hydropower plant and one “experienced” group that had migrated through the fish passage once before and were reintroduced to the migration choice.

## Materials and methods

### Study area

**Figure 1:**
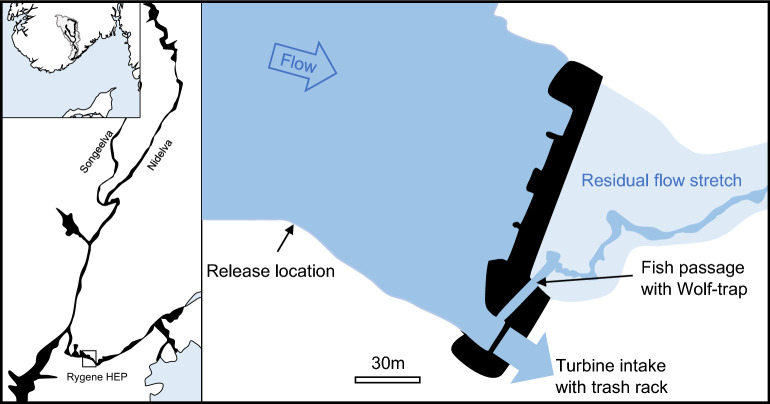
The watercourse of river Nidelva (upper) including the anadromous stretch with the Songeelva tributary (mid) and Rygene hydropower station with release location of tagged smolts, dam, turbine intake and fish passage.

The study was carried out in the river Nidelva, southern Norway (58.41540°N, 8.74242°E, Fig. 1). The river has a mean annual discharge of 110 m^3^ s^−1^ and the Atlantic salmon uses the lowermost 35 km as spawning and nursery habitat. Several tributaries are important habitats for salmonids in this river system, including the river Songeelva that intersects Nidelva at Froland, 25 km upstream the river mouth. Nidelva’s catchment is 4015 km^2^ and extensively regulated by 16 hydropower plants. The lowermost hydroelectric plant is a run-of-the river plant producing 55 MW. It is situated at Rygene, 9.4 km upstream from the river mouth. The experiments of the current study were undertaken at this power plant. During smolt migration in spring, water (5 m^3^ s^−1^) is released through a surface fish passage to aid the salmonid smolts pass the facility`s single Kaplan turbine intake^32^. The fish passage is located perpendicular to the approaching flow on the eastern side of the submerged intake trash rack (15 m × 9 m). The fish passage is 4 m wide and opened to a 0.7 m depth during the study period. The bar spacing in the trash rack is 80 mm, and thus large enough for smolts to pass. When in the hydropower forebay, the smolts are thus faced with a choice of two different migration alternatives with very different characteristics: one being a submerged, dark and fenced turbine intake and the other a small surface fish passage channel. The water velocity in front of the trash rack varies with river discharge. At turbine maximum capacity (170 m^3^ s^−1^) the mean velocity is 1.2 ms^−1^ and decrease to 0.5 ms^−1^ when turbine discharge is 70 m^3^ s^−1^.

### Fish sampling and tagging

Downstream migrating wild Atlantic salmon smolts were caught at two locations during the smolt migration period; in a Wolf-trap placed in the fish passage at Rygene and in the tributary Songeelva using a modified fyke-net (Table 1). Traps were emptied every morning. Smolts captured in Songeelva were transported by car to the Rygene hydropower station. The smolts were anesthetized with MS222 (Metomidate) (2 mg/l) before being tagged internally with passive integrated transponder tags (23 mm, half duplex, Oregon RFID). The tag was inserted through a small incision made ventrally between the posterior tip of the pectoral fin and the anterior point of the pelvic girdle. Several studies show that PIT-tagging has low likelihood of imposing mortality ^33,34^. Smolts were measured for total length (mm). Permission to catch Atlantic salmon smolt in River Nidelva was granted by the County Governor of Agder. PIT-tagging and behaviour assays of smolt were approved by Norwegian Animal Research Authority, NARA (FOTS ID 15463) and in accordance with national laws for experiments using live animals. All personnel involved in the tagging and handling of fish were trained and familiar with FELASA guidelines. One smolt died prior to the *willingness to leave a familiar area* assay (see below)*,* while two smolts jumped out of the aquarium during the *basal locomotion* assay. These three smolts were excluded from the analyses.

**Table 1:** Number of PIT-tagged Atlantic salmon smolts released upstream Rygene hydropower plant during 2018 smolt migration period. Experienced smolts were caught in the trap at Rygene fish passage while naive smolts were caught in Songeelva tributary. All smolts that went through behavioural assays were naive, caught in Songeelva tributary.

Release date	Consistency test		Behavioural assay
	Experienced	Naive	
04.05.2018			1
05.05.2018		2	
06.05.2018	1	1	4
07.05.2018		1	16
08.05.2018	20	55	27
09.05.2018	24	2	19
10.05.2018	85	14	20
11.05.2018	93	14	34
12.05.2018		87	5
13.05.2018			10
14.05.2018	52	166	5
15.05.2018	31	43	24
16.05.2018		5	1
17.05.2018		1	
18.05.2018		2	18
	306	393	184

### Behavioural assay

Behaviour traits were scored in each of the following contexts: A basal locomotor activity (adapted from previous studies^35–37^), response to net restrain (adapted from previous studies^37,38^) and willingness to leave a familiar area (adapted from previous studies ^38–40^). These assays were chosen because they have been used in previous studies to characterize important aspects of behaviour in fish, such as basal activity pattern, escape response and risk-taking behaviour^37–39^.

#### Basal locomotion

After a 24 h post-tagging recovery period, smolts were netted from the holding tank and inserted individually into visually isolated observation aquariums (25, 15, 20 cm L, W, D) standing on a UV-table. After 10 min of acclimatisation in the aquariums, fish behaviour was recorded in total darkness by video cameras with UV-filters for 20 min, and the analysed for swimming distance by EthoVision XT (Noldus, Version 11).

#### Net restrain

After the locomotion assay, fish were gently netted out of the aquarium. The net was placed above water on a support stand. While in the net, smolt escape attempts was recorded by video camera for 10 sec. An escape attempt was defined as a 45 ˚ tail beat. Individual tail beats were counted subsequently by running the video in slow motion.

#### Willingness to leave a familiar area

Each day, individually behaviour-scored fish were placed in a white semi-transparent tank (1x1x1 m) at the riverside. The tank was supplied with flow-through river water. A dark tube (10 cm diameter) drained water from the surface of the tank back into the river. After one night of acclimation to the tank environment, a net was removed from the surface tube and smolts could swim back into the River Nidelva 100 m upstream the hydropower plant. Individuals leaving the tank during the next 32 h (termed “leavers”) were registered in a PIT-antenna mounted on the escape tube. Remaining individuals (after 32 h) (termed “stayers”) were manually PIT-scanned for identification. Statistical analysis was done by comparing these two groups. To allow assays to be initiated every day, the assay was performed in duplicate tanks.

### Consistency of migration route choice

When testing for consistency of migration route choice, we compared the migration route choice of smolts that had already selected the fish passage once before (experienced), with smolts that had never seen a hydropower plant (naive). Experienced fish were retrieved from the Wolf-trap that is in the fish passage at Rygene hydropower plant. We do not have the same opportunity for retrieving individuals that originally used the turbine route. Smolts caught in the Songeelva tributary are individuals that were naive to the hydropower water intake. In the following we term this “load experience” with a naive and experienced group. Smolts from the two locations were PIT-tagged and placed in the same holding tank supplied with flow through river water. After one night, smolts were released in the hydropower forebay 100 m upstream of the water intake and fish passage at Rygene.

### Recapture of tagged smolts

PIT-tagged smolts that had been subjected to behavioural scoring and consistency test were captured in a Wolf-trap if they used the fish passage. The Wolf-trap covered the entire water column in the fish passage channel with bar spacing of 10 mm. The Wolf-trap was deployed from 26.04 to 28.05.2018. Tagged individuals were identified using a handheld PIT-reader. Turbine migrants could not be detected using this technology due to methodological limitations of PIT-antenna size and placement in such high-discharge / high-current tail-race areas. Non-recaptured smolts were therefore assumed to be turbine migrants. The short distance from the release point to the water intake makes it unlikely that smolts could be predated or shed the tag and thus be wrongly assigned to the turbine migration group. There is no other migration route past the dam except turbine intake or fish passage. The fish passage was closed at the end of May, thus 10 days after the last smolt release group.

### Statistical analysis

The statistical software R ^41^ was used for data inspection and statistical analyses. In order to check for correlations between behavioural assay scores linear models (lm)were fitted. The probability of choosing the fish passage option was estimated by fitting candidate generalized linear models (GLM) by including the following potential predictor variables: the ratio of water discharge through the fish passage to the flow passing through the turbine intake (Relative fish passage discharge, Q_rel_ = Q_fish passage_/Q_turbine_), total river discharge (Q = water discharge (m^3^/sec)), fish length (mm), locomotor activity (the rates of movements recorded in the assay (cm/min)), the net restrain assay (number of escape attempts) and willingness to leave a familiar environment (0 = leavers; 1 = stayers).

To test for consistency of migration route choice at the hydropower forebay we fitted candidate GLMs with catch location (Songeelva tributary: “naive” and Rygene fish passage: “experienced”), fish length and relative fish passage discharge as predictor variables. The logit link function was used for linearization of the binomial response in both GLMs (0 = not recaptured; 1 = recaptured in fish passage). Model selection was based on corrected Akaike's information criterion (AICc;^42,43^) using the MuMIn library (Baron^44^). In cases where candidate models attained AICc scores below 2 (e.g., Burnham and Anderson^45^), model averaging was undertaken and estimates of variable importance estimated to aid the multi-model inferences.

## Results

The Atlantic salmon smolt migration period commenced on 3 May in the Songeelva tributary and ended 15 May. The median migration date was 3 days earlier in the Songeelva tributary than in the main river at Rygene, although there was not a statistically significant difference in catch trajectories between the two sites (*p* > 0.05 two-sample Kolmogorov–Smirnov test). Relative fish passage discharge was on average 4.2 ± 1.23% (± SD) of the volume passing through the turbine intake during the smolt migration period. The Songeelva smolt were on average 131 ± 9.8 mm long (± SD).

The smolts had an average swimming speed of 52.14 ± 57.15 cm/min (± SD) during the basal locomotor activity assay, and there was a positive relationship between the smolt activity in the first and final 10 min of the assay (*p* < 0.001) but no correlation with the net restraining assay results (*p* > 0.05). Number of escape attempts during the 10 s net restraining assay were on average 13.6 ± 8.4 (± SD). There were no significant statistical differences in the escape attempts nor locomotor activity between smolts that left or stayed in the familiar environment (*p* > 0.05). In total, 33.3% of the smolts left the familiar environment before the assays were terminated. When back in the river, 63 smolts were recorded in the fish passage, while the remainder 121 individuals were assumed to be turbine migrants. There was no correlation between fish length and the three behavioural assays (*p* > 0.05).

**Figure 2:**
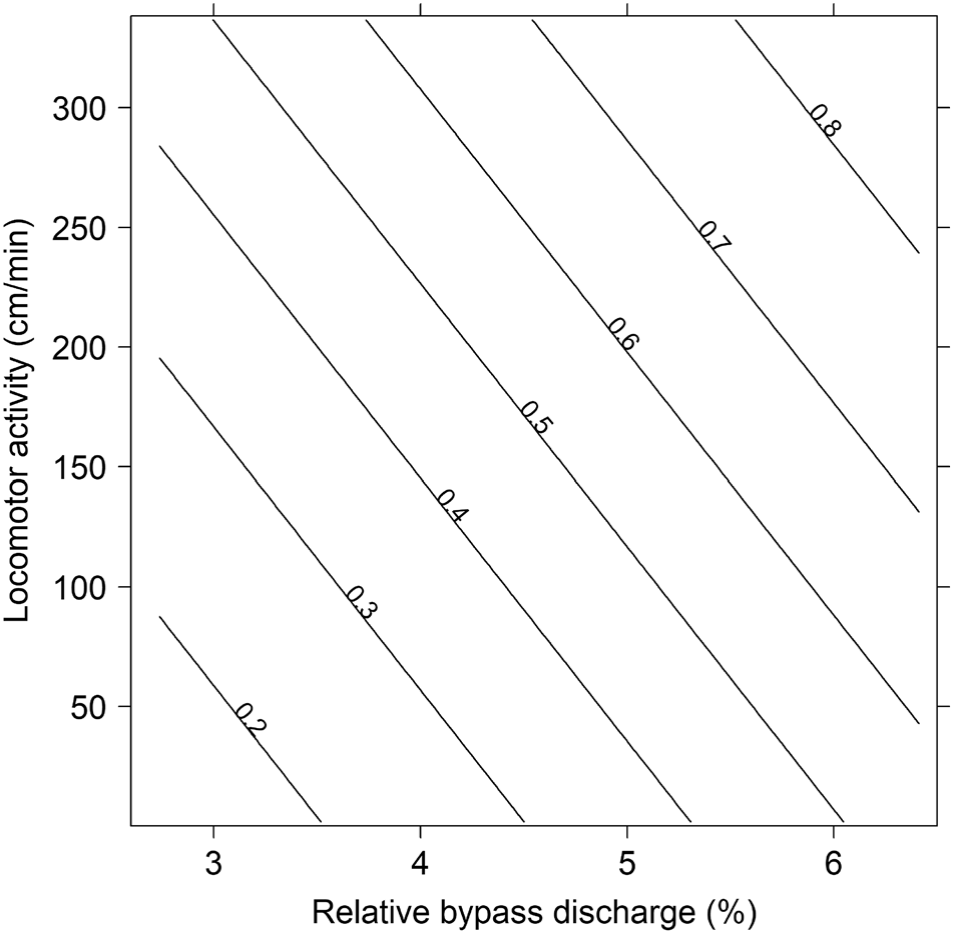
Predicted fish passage migration probability for Atlantic salmon smolts at Rygene hydropower plant as a function of locomotor activity (distanced swam per min during the 20-min basal locomotion activity assay) and percent discharge allocated to the fish passage in relation to the turbine intake. Probability predictions, displayed as isolines, were derived from the most supported binomial GLM, as reported in Table 2.

To investigate the strength and direction of key factors’ effects on fish passage migration probability, environmental and individual variables (including the behavioural assay traits) were incorporated in candidate GLMs. AICc-based model selection revealed highest support (21% of the support among all candidate models, Table S1) for an additive model including the ratio of discharge passing through the fish passage to that in the turbine intake (relative fish passage discharge %) and activity (i.e., Pr [fish passage migration] = Activity + Q_rel_, Table 2, Fig. 2). In addition to the top model, three candidate models attained ΔAICc-values below 2. The relative fish passage discharge effect was included all top-four models and activity in three of them attaining importance estimates of 1.00 and 0.60, respectively. Willingness to leave a familiar environment was included as a predictor in two of the four top-ranked models, with an estimated importance of 0.49. The top model predicted, based on model averaged parameter estimates, highest probability of fish-passage migration when a high amount of water was released in the fish passage for individuals that had a high basal locomotor activity (Fig. 2).

**Table 2:** Logit-parameter estimates (modell averaged) and corresponding likelihood-ratio test statistics for the most supported GLM fitted to predict fish passage probabilities in PIT-tagged Atlantic salmon smolts from the Nidelva tributary Songeelva. Q_rel_ (Relative fish passage discharge) = Discharge in the fish passage/discharge in the turbine tunel, Activity = The distance smolt swam in the aquarium during the 20 min trail.

Parameter estimates			LR-test statistics			
Term	Coeff	SE	Effect	df	χ^2^	*p*
Intercept	− 3.1886	0.7943	*Q*_*rel*_	1	17.83	< 0.0001
*Activity*	0.0052	0.0031	*Activity*	1	7.12	0.0076
*Q*_*rel*_	0.5013	0.2094

**Figure 3:**
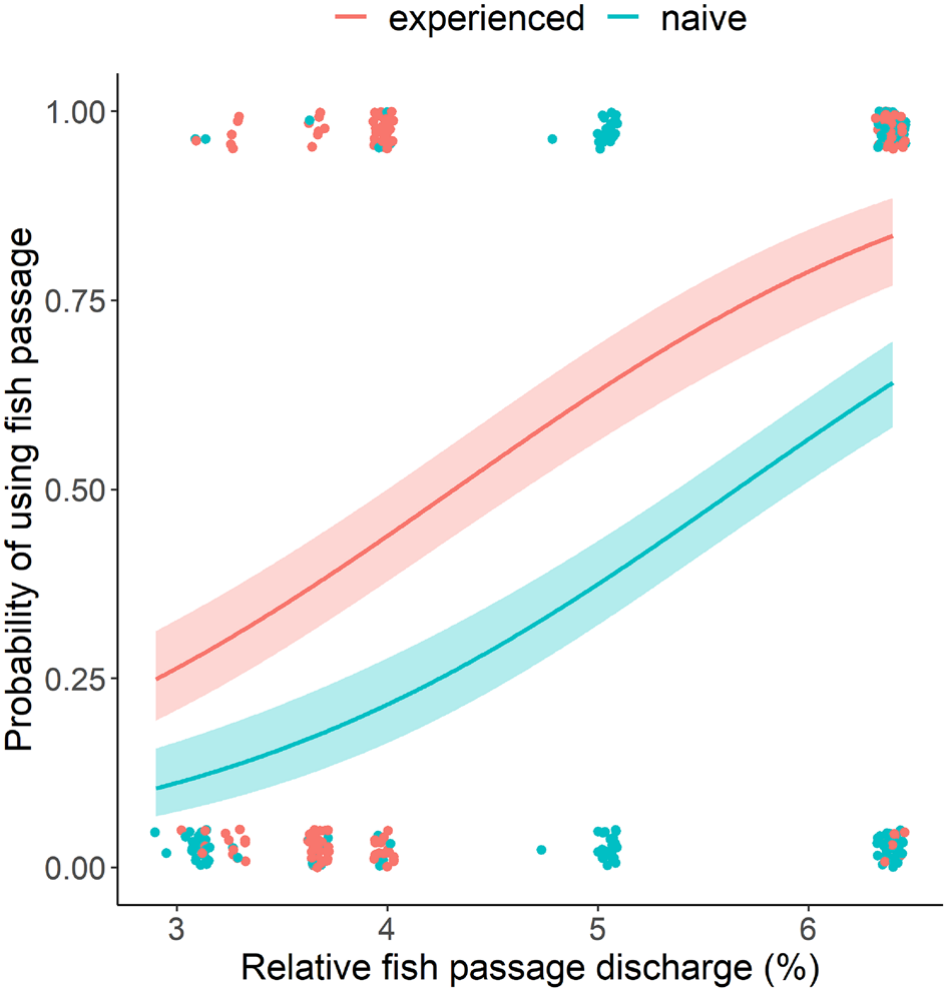
Predicted fish passage migration probability for experienced (red line) and naive (blue line) Atlantic salmon smolts at Rygene hydropower plant as a function of water allocation, as derived from the selected binomial GLM reported in Table 3. Shaded areas represent 95% confidence bounds including the distribution of individual fish passage- (y = 1) and turbine migrants (y = 0) at different discharge regimes (the observations have been x–y-jittered to reveal overlapping data).

155 out of 306 experienced smolts (51%) showed consistency in migration route choice and were recaptured in the fish passage a second time, while 183 out of 393 naive smolts (47%) used the fish passage. AICc-based model selection revealed highest support in the data for an additive effect of relative fish passage discharge and load experienced on the probability of fish passage migration at the hydropower plant (i.e., Pr [fish passage migration] = Q_rel_ + load experience; Table 3, Fig. 3). This model attained 66% of the AICc-support and an AICc‐score 1.36 lower than the second-most supported model (load experience + Q_rel_ + fish length; 34% AICc support) (Supplementary Information Table S3). Because model averaging based on the two top models (that attained almost 100% of the AICc support) did not change the initial parameter estimates, this step was not implemented in the model prediction process. The selected model predicted fish to have the highest probability of using the fish passage when a high amount of water was released through it, and the probability was higher for experienced than naive smolt (Fig. 3).

**Table 3:** Logit-parameter estimates and corresponding likelihood-ratio test statistics for the best supported GLM fitted to predict fish passage probabilities for fish passage experienced and fish passage naive PIT-tagged Atlantic salmon smolts at Rygene hydropower plant. Q_rel_ (Relative fish passage discharge) = Discharge in the fish passage/discharge in the turbine intake, Load experience = Fish passage naive (catch location: Songeelva) and fish passage experienced (catch location: Rygene fish passage).

Parameter estimates			LR-test statistics			
Term	Coeff	SE	Effect	df	χ^2^	*p*
Intercept	− 3.367	0.352	*Load (Naive)*	1	1.15	< 0.001
*Load (Naive)*	− 1.045	0.201	*Q*_*rel*_	1	126.69	< 0.001
*Q*_*rel*_	0.780	0.078

## Discussion

In this study, we showed that individual differences in migration route choice at a hydropower plant were a consistent trait, and associated with relative fish passage discharge and contrasting individual behavioural profiles in migrating Atlantic salmon smolts.

Allocating relatively more flow to the fish passage facility increased the number of fish using the fish passage. This finding is in accordance with previous studies^32,46^ and several national fish passage guidelines^47–49^. This study’s hydropower site may therefore, at least in theory, use its ability to control water flow to the fish bypass to optimize discharge levels to maximize smolt usage throughout the smolt run period. This would increase guidance efficiency without spilling unnecessary amounts of water that could be used for electricity production.

Smolt with a high basal activity pattern had higher probability of using the fish passage than smolt with lower activity in this study. Generally, activity is widely used as a personality proxy in animal behavioural research ^50^. Accordingly, field studies of wild salmonid document repeatability of movement patterns over time and across different contexts ^51^. In a previous study in the river Storelva, Haraldstad ^28^ showed that some smolts migrated into the turbine intake almost instantly, while others hesitated and stayed in the forebay for days until a suitable alternative migration route became available. Moreover, it was hypothesized that the turbine migrants and hesitating individuals represented different behaviour types. At hydropower facilities in general and in particular at our study site, it is important to consider that the smolts are faced with a choice of two different migration alternatives with very different appearances: One being a dark, submerged and fenced tunnel and the other a small, surface-layer bypass channel. The present study demonstrated that the route choice is linked at least in part to individual variation in basal activity. This lends support to the hypothesis that differences in behavioural phenotypes affect route preferences of Atlantic salmon smolt at hydropower plant water intakes.

We found individuals with former fish passage experience had, under similar discharge conditions, a significantly higher probability of choosing fish passage facilities as opposed to turbine intakes compared to their naive counterparts. This finding demonstrated that the route choice is a partly consistent trait and strengthens the hypothesis that the migration route preferences are partly based on individual trait variation, and not environmental aspects alone. An alternative explanation is that experienced smolts learned from their previous choice ^52^ and altered their behaviour. Our experimental set-up was not designed to distinguish between learning and personalities. Furthermore, learning and personalities may be linked, as cognitive abilities have been suggested to be linked to personality traits in fish ^53^. Given the significantly higher survival for fish passage migrants than turbine migrants ^8^ and the longevity of hydropower facilities compared to salmon generation times, this suggests the possibility of hydropower-induced evolutionary responses on traits associated with the migration route choice, provided sufficient additive genetic variance for these traits is present and that this variance is correlated with life-time fitness ^54^. Clearly, a smolt does not only face behavioural selection while descending the river, and changes favoured to survive hydropower systems may be countered by other selection pressures later in life. This makes it hard to predict what the ultimate outcome of these combined selection factors will favour in the longer term. As a first step our data suggests that mitigation measures at hydropower plants aimed at countering turbine mortality potentially will induce new selection regimes.

Generally, behaviour traits tend to disassociate during ontogenetic shifts ^55^. Such a disassociation phenomenon may explain why just basal activity pattern seemed to impact route choice for smolts in this study, where other behaviour traits did not. This finding indicates that previously reported relations between responses in the different behavioural assays ^37,38,56^, have weakened in our groups of fish during the smolt stage. This opens up the possibility that correlations among behavioural traits measured during the parr stage are behavioural syndromes that could predict route choices during smolt descent. However, If the same group of fish get scored in the same behavioural assays during the smolt stage, the grouping into behavioural syndromes may no longer be possible, and route choice may just be possible to demonstrate for a few individual behaviour traits ^57^. Further studies are needed to clarify how physiological and behavioural changes associated with smolting affect an individual’s trait association and how these, in turn, are affected by selection in anthropogenically altered environments.

Allocating relative more flow to the fish passage facility increased the number of fish using the fish passage in both experiments and this is in accordance with several other studies ^32,46^. Still, smolts with low basal activity had a lower probability of using the fish passage than their more active counterparts over the range of flow regimes during our experiment. Most measures used to get migratory fish to use bypasses depend on certain fish behaviour responses to function. Responses that result in fish preferentially choosing turbine intakes over fish passages will thus potentially act as selective agent imparting higher mortality and selecting against the choice of high flow pathways at some hydropower sites. However, personality differences are important in wild populations for maintaining stability, resilience, and persistence and their genetic component makes them an important dimension of biodiversity ^58,59^. In fisheries, there is an increasing body of literature showing how anthropogenic selection regimes transform the adaptive landscape and induce selection on behavioural traits ^60–62^. Given the lessons learned from these studies, selection on behavioural traits may be expected to act on fish living in hydropower regulated ecosystems, leading to an altered evolutionary pattern followed by a reduction in behaviour and corresponding genetic diversity. The present study highlights the importance of non-selective fish passages at hydropower plants. Specifically, implementing small-spaced trash racks will prohibit descending Atlantic salmon smolts from entering the turbine intake and help protect the population behavioural diversity.

## Supplementary Information

Supplementary Information.
