# Supplementary material for: Hydropower-induced selection of behavioural traits in Atlantic salmon (Salmo salar)
Source: Sci Rep. 2021 Aug 12;11:16444. doi: 10.1038/s41598-021-95952-1 (PMC8360942; doi:10.1038/s41598-021-95952-1)
Supplement: Supplementary file 1 — Supplementary Information. [file 41598_2021_95952_MOESM1_ESM.docx]

## Supplementary Information

**Table S1**. Model selection table for the 5 most supported candidate GLM-models (ΔAICc <2) fitted to predicted fish passage migration probability for Atlantic salmon smolts at Rygene. K = number of parameters fitted, AICc = corrected Akaike’s information criterion, ΔAICc = difference between a model’s AICc value and the most supported model, AICcWt = the relative AICc support of the model, *LL*=the model log likelihood. Activity= The rates of movements recorded in the basal locomotion assay (cm/min), Q_rel_ = Relative fish passage discharge (the ratio of water discharge through the fish passage to the flow passing through the turbine intake), WLFE= Willingness to leave a familiar environment (0=leavers; 1=stayers).

| Model structure | K | AICc | ΔAICc | AICcWt | Cum.Wt | *LL* |
| --- | --- | --- | --- | --- | --- | --- |
| Activity + Q_rel_ | 3 | 221.78 | 0 | 0.21 | 0.21 | -107.82 |
| Q_rel_ | 2 | 222.59 | 0.82 | 0.14 | 0.35 | -109.26 |
| Q_rel_ + Activity + WLFE | 4 | 222.81 | 1.03 | 0.13 | 0.48 | -107.3 |
| Activity + WLFE* Q_rel_ | 5 | 223.76 | 1.98 | 0.08 | 0.56 | -106.72 |
| Activity * Q_rel_ | 4 | 223.83 | 2.05 | 0.08 | 0.64 | -107.81 |
|  |  |  |  |  |  |  |

**Table S2**. Model-averaged logit-parameter estimates for the top four models provided in Table S1, fitted to predicted fish passage migration probability for Atlantic salmon smolts at Rygene. Rows with bold-faced letters represent the top model. Activity= The rates of movements recorded in the basal locomotion assay (cm/min), Q_rel_ = Relative fish passage discharge (the ratio of water discharge through the fish passage to the flow passing through the turbine intake), WLFE= Willingness to leave a familiar environment (0=leavers; 1=stayers)

| Term | Estimate | SE | Adj SE | z | p |
| --- | --- | --- | --- | --- | --- |
| **Intercept** | **-3.1886** | **0.7901** | **0.7943** | **4.0140** | **<0.0001** |
| **Activity** | **0.0026** | **0.0015** | **0.0015** | **1.6900** | **0.0911** |
| **Q_rel_** | **0.5013** | **0.2084** | **0.2094** | **2.3950** | **0.0166** |
| WLFE | -0.1497 | 1.1506 | 1.1554 | 0.1300 | 0.8969 |
| WLFE*Q_rel_ | 0.3659 | 0.3431 | 0.3453 | 1.0600 | 0.2893 |

**Table S3.** Model selection table for the 6 most supported candidate GLM-models fitted to predicted fish passage migration probability for Atlantic salmon smolts at Rygene. K = number of parameters fitted, AICc = corrected Akaike’s information criterion, ΔAICc = difference between a model’s AICc value and the most supported model, AICcWt = the relative AICc support of the model, *LL*=the model log likelihood. Load experience= Smolts former experience with a hydropower plant (“naive” or “experienced”), Q_rel_ = Relative fish passage discharge (the ratio of water discharge through the fish passage to the flow passing through the turbine intake), Length = Smolt total length (mm)

| Model structure | K | AICc | ΔAICc | AICcWt | Cum.Wt | *LL* |
| --- | --- | --- | --- | --- | --- | --- |
| Load experience + Q_rel_ | 3 | 846.45 | 0 | 0.66 | 0.66 | -420.21 |
| Load experience + Q_rel_ + Length | 4 | 847.81 | 1.36 | 0.34 | 1.00 | -419.88 |
| Q_rel_ + Length | 3 | 874.30 | 27.85 | 0.00 | 1.00 | -434.13 |
| Q_rel_ | 2 | 874.52 | 28.07 | 0.00 | 1.00 | -435.25 |
| Length | 2 | 970.54 | 124.09 | 0.00 | 1.00 | -483.26 |
| Load experience | 2 | 971.13 | 124.68 | 0.00 | 1.00 | -483.56 |
